# Supplementary material for: Research on multiple bubbles in China’s multi-level stock market
Source: PLoS One. 2021 Aug 2;16(8):e0255476. doi: 10.1371/journal.pone.0255476 (PMC8328329; doi:10.1371/journal.pone.0255476)
Supplement: Attachment — Submitted filename: Response to Reviewers.docx [file pone.0255476.s005.docx]

Dear Dr. Yang:

Thank you for your efficient work in processing our manuscript entitled “Research on multiple bubbles in China's multi-level stock market” (Manuscript No: PONE-D-20-36507). After we have carefully read the comments from the reviewers, we realize that the major merits of our work were not fully identified or recognized by the reviewers. Here we would respond to the reviewers’ comments (the replies are highlighted in blue).

Reviewer #1:

1.The topic is not interesting.

Response：As China becomes the second largest economy in the world, research on China's capital market has become increasingly important. Besides, as a representative emerging market, research on China's capital market can help people understand the characteristics of emerging markets. China's stock market is characterized by a dominance of inexperienced individual investors, binding short‐sales constraints (lifted only in 2011), a small asset float (before the split‐share reform of 2005–2006) and heavy share turnover despite high transaction costs. Individual investors in China's stock market are less informed and more subject to behavioral biases than institutional investors. All these factors make very likely the presence of active speculative behavior in Chinese stock market. Therefore, our work is meaningful.

Most researches in China only adopt price series to detect capital market bubble without considering dividends. It does not conform to the specification of test model and cannot be theoretically supported. For example, Company A's stock price is 100 Yuan per share, cash dividend is 10 Yuan per share, while company B's stock price is 10 Yuan per share, cash dividend is 1 cent per share. At this time, there are more likely bubbles in B's share price. If only considering the price 100>10, it is biased to say there is a bubble in company A's stock price. Different from them, we select the ratios of the natural logarithm of real stock prices to the natural logarithm of real dividends to conduct tests from perspective of combination of stock price and dividend. Therefore, our work is different and interesting.

2. Critical values should be generated to account for the presence of non-stationary volatility. Hence, all the analysis in this paper is flawed.

Response：Recursive explosive‐root (vs random walk) tests (Phillips, Shi, & Yu, 2015a,b) with a unit root (market fundamental) null and an explosive (bubble) alternative enable researchers to detect the rise and collapse of bubbles on a given asset market. It is shown by Phillips, Shi, and Yu (2015a) that the PSY method outperforms the other recursive methods. The PSY procedure aims to detect the local explosive dynamics of speculative bubbles. The principle and reliability of this method are described in detail in articles “EXPLOSIVE BEHAVIOR IN THE 1990s NASDAQ: WHEN DID EXUBERANCE ESCALATE ASSET VALUES?*” (Phillips, Wu, & Yu, 2011) and “TESTING FOR MULTIPLE BUBBLES: HISTORICAL EPISODES OF EXUBERANCE AND COLLAPSE IN THE S&P 500∗” (Phillips, Shi, & Yu, 2015a). The statistical analysis in our work are appropriate and rigorous.

3. This paper has numerous grammar and language issues.

Response：We have found English professionals to read our article and made a lot of grammatical changes. All the changes have been marked red in the revised manuscript. The modifications are as follows.

(1) Page 2, line 24-26:

ORIGINAL: Understanding bubble phenomenon and dating the origination and termination points in real time can provide an early warning diagnosis for financial bubbles and help regulatory authorities to control it and maintain market order.

REVISED: Understanding bubble phenomenon and dating the period of bubbles in real time can provide an early warning diagnosis for financial bubbles and help regulatory authorities to control it and maintain market order.

(2) Page 3, line 55-57:

ORIGINAL: Rational bubble theory assumes that the actual price of an asset is equal to the present value of relevant fundamentals and the bubble component that is expected to grow at the real interest rate, and that investors have rational expectations.

REVISED: Rational bubble theory assumes that the actual price of an asset is equal to the sum of the present value of relevant fundamentals and the bubble component which is expected to grow at the real interest rate, meanwhile the investors have rational expectations.

(3) Page 3, line 57-59:

ORIGINAL: Early rational bubble test based on the assume that bubble was linear, namely, if there was a bubble, it would always exist, and would not burst or start again.

REVISED: Early rational bubble test based on the assume that the bubble was linear, which meant that the bubble would always exist and would not burst or start again.

(4) Page 4, line 69-70:

ORIGINAL: In addition, if there is a relatively short-term bubble in a long time series of data, it may not correctly identify the presence of bubble.

REVISED: In addition, if there is a relatively short-term bubble in a long time series of data, the existence of this bubble may not be correctly recognized.

(5) Page 4, line 71:

ORIGINAL: The above methods have imposed very little structure on bubbles. Actually, bubbles have certain theoretical properties can be clearly used for detection.

REVISED: However, there are certain theoretical properties that can be clearly used for bubbles detection.

(6) Page 4, line 74-77:

ORIGINAL: Diba and Grossman [12-13] observed that rational bubble could not start (or restart); therefore, if it exists now, it must exist on the first day of trading and always exist. If the bubble "pops", it must collapse to zero because of the lack of arbitrage opportunities and the impossibility of negative prices.

REVISED: Diba and Grossman [12-13] observed that rational bubble could not start (or restart); therefore, if there is a bubble, it must exist on the first day of trading and always exist. If the bubble "pops", the bubble must collapse to zero due to the lack of arbitrage opportunities and the impossibility of negative prices.

(7) Page 5, line 91-92:

ORIGINAL: But the Markov model is computationally expensive. And, in some cases, the asymptotic distribution of unit root test statistic calculated by this model is impossible to analyze.

REVISED: However, the Markov model is computationally expensive, and the asymptotic distribution of unit root test statistic calculated by this model is impossible to analyze in some cases.

(8) Page 5, line 93-95:

ORIGINAL: In recent years, the breakthrough of periodical bubble test is that they apply right-tailed DF tests of unit root null hypothesis against explosive autoregression alternative hypothesis to relevant series in levels only.

REVISED: In recent years, the breakthrough of the periodical bubble test is that the right-tailed DF tests of unit root null hypothesis against explosive autoregression alternative hypothesis is applied to relevant series in levels only.

(9) Page 7, line 134-138:

ORIGINAL: The rest of this paper is organized as follows. Section 2 discusses the basic model specification of bubble test and the principles of right-tail recursive ADF test, including SADF, GSADF and BSADF. Section 3 introduces the sample processing, and provides related preliminary data analysis. The empirical bubble test results and result analysis are presented in Section 4. Section 5 concludes this paper.

REVISED: The rest of this paper is organized as follows. In Section 2, the basic model specification of bubble test and the principle of right-tail recursive ADF test are discussed, including SADF, GSADF and BSADF. In Section 3, the sample processing is introduced and the relevant preliminary data analysis is provided. The empirical bubble test results and result analysis are introduced in Section 4. In Section 5, the full text is summarized and conclusions are drawn.

(10) Page 10, line 201-202:

ORIGINAL: So the null hypothesis and the alternative hypothesis are respectively:

REVISED: Therefore, the null hypothesis and the alternative hypothesis are:

(11) Page 25, line 477-478:

ORIGINAL: By contrast, China's market fluctuates frequently, not only does it have multiple bubbles, but each time the bubble starts and bursts rapidly.

REVISED: In contrast, the China's market fluctuates frequently, not only with multiple bubbles, but also with short period.

(12) Page 25, line 492-495:

ORIGINAL: Compared with institutional investors, small and medium-sized individual investors lack professional knowledge and investment skills and have a serious herd mentality. They are more concerned about other people's investment behavior and blindly follow the trend.

REVISED: Compared with institutional investors, small and medium individual investors lack professional knowledge and investment skills, and have a serious herd mentality. They pay more attention to the investment behavior of others and blindly follow suit.

4. Have the authors made all data underlying the findings in their manuscript fully available?

Reviewer #1: No

Response：We made all data underlying the findings in our manuscript fully available. We provided the data in supporting information of our manuscript.

We understand that the misunderstanding might be caused by the unclear description in our manuscript, but we believe that the results are of merit and the paper is potentially publishable in the journal. Therefore, we would be most grateful if you could re-consider our work and give us a second opportunity. Thank you very much for your patience and understanding. If you have any question about this paper, please don’t hesitate to let me know. Looking forward to hearing from you.

Kind regards,

Ge Li, Ph.D candidate

Corresponding author: Ming Xiao, Ph.D

E-mail address: blue_ridge111@yahoo.com
