# Supplementary material for: Research on multiple bubbles in China’s multi-level stock market
Source: PLoS One. 2021 Aug 2;16(8):e0255476. doi: 10.1371/journal.pone.0255476 (PMC8328329; doi:10.1371/journal.pone.0255476)
Supplement: Attachment — Submitted filename: Response to Reviewers.docx [file pone.0255476.s006.docx]

To academic editor and reviewers,

*PLOS ONE*

Dear Prof. J E. Trinidad Segovia and Reviewers:

Thank you for giving us a chance to resubmit the paper and your constructive comments on our manuscript entitled "Research on multiple bubbles in China's multi-level stock market" (ID: PONE-D-20-36507R1).

Those comments are very helpful for revising and improving our paper. We have studied the comments carefully and made corrections which we hope meet with approval. Efforts were also made to correct the grammatical mistakes in the manuscript. We mark all the changes in red in the revised manuscript with track changes. Attached please find our revised manuscript. Listed below are our point-by-point responses to the editor and reviewers’ comments (the replies are highlighted in blue).

Responds to academic editor:

**Comment 1:** Please review your reference list to ensure that it is complete and correct. If you have cited papers that have been retracted, please include the rationale for doing so in the manuscript text, or remove these references and replace them with relevant current references. Any changes to the reference list should be mentioned in the rebuttal letter that accompanies your revised manuscript. If you need to cite a retracted article, indicate the article’s retracted status in the References list and also include a citation and full reference for the retraction notice.

**Response:** We have reviewed our reference list; all the reference are complete and correct. We didn’t cite papers that have been retracted. We made some changes in our references:

- we deleted reference [16]: Van Norden S. Regime switching as a test for exchange rate bubbles. J. Appl. Econom. 1996; 11(3): 219-251. doi: 10.1002/(SICI)1099-1255(199605)11:3<219::AID-JAE394>3.0.CO;2-S.
- we added references:

[33].	Sethi SP. When does the share price equal the present value of future dividends? Econ. Theory. 1996; 8(2): 307-319. doi: 10.1007/BF01211820.

[34].	Sethi SP, Derzko NA, Lehoczky JP. A stochastic extension of the Miller-Modigliani framework. Math. Finance. 1991; 1(4): 57-76. doi: 10.1111/j.1467-9965.1991.tb00019.x.

[35].	Sethi SP, Derzko NA, Lehoczky JP. Mathematical analysis of the Miller-Modigliani theory. Operations Res. Lett. 1982; 1(4): 148-152. doi: 10.1016/0167-6377(82)90018-9.

[37]. Rambaud SC. Arbitrage theory with state-price deflators. Stoch. Models. 2013; 29(3): 306-327. doi: 10.1080/15326349.2013.808902.

[41]. Fernández-Martínez M, Sánchez-Granero MA, Muñoz Torrecillas MJ, Mckelvey B. A comparison of three Hurst exponent approaches to predict nascent bubbles in S&P500 stocks. Fractals. 2017; 25(01): 1750006-105. doi: 10.1142/S0218348X17500062.

The detail changes of our references are elaborated in the following responds to the reviewers.

**Comment 2:** When submitting your revision, we need you to address these additional requirements. Please ensure that your manuscript meets PLOS ONE's style requirements, including those for file naming.

**Response:** After reading the PLOS ONE style templates carefully, we ensure that our manuscript meets PLOS ONE's style requirements, including those for file naming.

**Modification 1:** We have added a second affiliation to co-author Xionghui Yang. The detailed information of this affiliation is added in page 1, line 5 of the revised manuscript: CITIC Group Corporation, Beijing, Beijing, People's Republic of China.

**Modification 1:** We have renumbered the equation after equation (10). The number of equation in page 14, line 279 is changed from (12) to (11); and the equation in the next line is changed from (13) to (12). The number of equation in page 15, line 295 is changed from (14) to (13). We are very sorry for our carelessness.

Responds to the reviewer’s comments:

Reviewer #3:

**Comment 1:** The paper contains numerous grammatical mistakes which should be immediately fixed (the list is not exhaustive):

* Line 26: Change "test" to "tests".

**Response:** According to the reviewer’s comment, we have changed "test" to "tests" in line 26. Furthermore, we have fixed the similar mistakes in the paper.

* Line 29: Change "Different from" to "Unlike".

**Response:** Thank you for your valuable advice. We have changed "Different from" to "Unlike" in line 29.

* Line 32: in the main board?

**Response:** We have changed “the main board” to “the Main-Board Market” in line 32. It refers to the traditional stock market, and is the main place for the issuance, listing and trading of securities in China.

* Line 34: credit easing cycle?

**Response:** We have changed “credit easing cycle” to “loose credit cycle”, which is the opposite of the tight credit cycle.

* Line 45: Change "bubble" to "bubbles".

**Response:** We have changed "bubble" to "bubbles" in line 45. We have also fixed the similar mistakes in the other part of the paper.

* Line 50: inflexibility? Please, define or clarify.

**Response:** In page 3 line 50, we changed “all showed the inflexibility of bubbles” to “are all the recognized bubble in the world”. The original statement is not accurate.

* Line 58: Change "assume" to "assumption".

**Response:** Thank you for your careful work. We have changed " assume " to " assumption " in line 58.

* Line 65: he explicitly put the bubble in alternative hypotheses? Please, explain.

**Response:** We have changed “he explicitly put the bubble in alternative hypotheses” to “the existence of bubbles was explicitly considered as alternative hypotheses for the first time.”. Detailed explanation: the alternative hypothesis of West's two-step tests was hypothesis that there was a bubble, in this test, the bubble was first explicitly considered as alternative hypotheses.

We are sorry for grammatical mistakes in this paper. We have carefully checked the manuscript vocabulary and grammar again. There are also some other revises in the revision:

(1)	on Page 2, line 30, " we select the ratios of the natural logarithm of real stock prices to the natural logarithm of real dividends instead of stock price indices to conduct tests. " has been revised as " the ratios of the real stock prices' natural logarithm to the real dividends' natural logarithm are used for our testing instead of stock price index. "

(2)	On Page 2, line 37, "can" has been revised as "not only".

(3)	On Page 2, line 38, "and" has been revised as "but".

(4)	On Page 3, line 61, "and" has been revised as ",".

(5)	On Page 3, line 63, " Marsh and Merton [8] suggested that variance bound tests would fail when dividends and stock prices were non-stationary. At the same time, there were small sample distortions. " has been revised as " Marsh and Merton [8] suggested that variance bound tests would fail when dividends and stock prices were non-stationary, and along with the small sample distortions. ".

(6) On Page 4, line 68, “rejecting null hypotheses too frequently” has been revised as " rejecting null hypotheses frequently ".

(7) On Page 5, line 93, “impossible” has been revised as " hard ".

(8) On Page 5, line 94, “In recent years, the breakthrough of the periodical bubble test is that the right-tailed DF tests of unit root null hypothesis against explosive autoregression alternative hypothesis is applied to relevant series in levels only.” has been revised as " In recent years, the breakthrough of the periodical bubbles test is that the right-tailed DF test is applied to relevant series. The null hypothesis is unit root process, and the alternative hypothesis is explosive autoregression process. ".

(9) On Page 5, line 101, “can” has been revised as " could ".

(10) On Page 6, line 106, “In addition, the estimates are consistent” has been revised as " , and the estimated points are consistent with the actual points ".

(11) On Page 6, line 118, “One simple way” has been revised as " A simpler method ".

(12) On Page 7, line 131, “ratios of the logarithm of real stock prices to the logarithm of real dividends rather than stock price indices are selected as target sequences,” has been revised as " in this paper, ratios of the real stock prices' natural logarithm to the real dividends' natural logarithm are selected as target sequences ".

(13) On Page 7, line 132, “times” has been added.

(14) On Page 10, line 199, “traditional ADF tests will confuse test results.” has been revised as " the results of traditional ADF test will be confusing".

(15) On Page11, line 217, “with” has been revised as "and".

(16) On Page11, line 227, “Further” has been revised as " Furthermore ".

(17) On Page12, line 244, “its test ability will be reduced” has been revised as " the applicability of this method will be reduced ".

(18) On Page13, line 256, “between BSADF and GSADF” has been added.

(19) On Page16, line 334, “this paper selects the ratios of the natural logarithm of real stock prices to the natural logarithm of real dividends” has been revised as "the ratios of the real stock prices' natural logarithm to the real dividends' natural logarithm (i.e. ln(p)/ln(d)) are selected ".

(20) On Page17, line 337, “dividends are less or even not, resulting in large fluctuations of the initial ln(p)/ln(d)” has been revised as " dividends are less, or even no dividends, which results in large fluctuations of the initial ln(p)/ln(d)".

(21) On Page17, line 344, Fig 4 Title has been revised from “Fig 4. The ratios of the natural logarithm of real stock prices to the natural logarithm of real dividends.” to " Fig 4. The ratios of the real stock prices' natural logarithm to the real dividends' natural logarithm.".

(22) On Page 18, line 358, “trend” has been added.

(23) On Page 18, line 369, “respectively” has been added.

(24) On Page 18, line 373, “times” has been added.

(25) On Page 18, line 378, “times” has been added.

(26) On Page 19, line 385, “it cannot be said” has been revised as " there is no evidence that ".

(27) On Page 20, line 395, “We can see” has been revised as " It is clear ".

(28) On Page 20, line 396, “this method has limited ability to identify multiple bubbles” has been revised as " the ability of this method to identify multiple bubbles is limited ".

(29) On Page 20, line 407, “In BSADF dating Results Fig 6, real price series are also plotted” has been revised as " The real price series are also plotted in Fig 6 ".

(30) On Page 22, line 427, “The longest is the bubble of 2013” has been revised as " The longest bubble appeared in 2013 ".

(31) On Page 22, line 430, “we can see” has been revised as " it is clear that ".

(32) On Page 24, line 458, “So, we can see bubbles in main board” has been revised as " Therefore, bubbles appeared in the Main-Board Market ".

(33) On Page 24, line 460, “there is a bubble in the middle of 2020 in three sectors, and as of August 28, 2020, it is not over” has been revised as " there are bubbles in all three sectors in mid-2020, and those bubbles have not yet ended as of August 28, 2020 ".

(34) On Page 24, line 461, “Why China's stock market soared this year despite many disasters? There must be a reason for this.” has been revised as " There must be a reason why China's stock market is still soaring this year in the troubled situation ".

(35) On Page 24, line 463, “The resumption of work and production by entities, especially small and medium-sized enterprises, is slow” has been revised as " The resumption of work and production is slow, especially for small and medium-sized enterprises ".

(36) On Page 27, line 528, “Different from the other researchers in China, we select the ratios of the natural logarithm of real stock prices to the natural logarithm of real dividends instead of stock price indices to conduct tests” has been revised as " Unlike the other researches in China, the ratios of the real stock prices' natural logarithm to the real dividends' natural logarithm are used for our testing instead of stock price index ".

(37) On Page 27, line 532, “First, GSADF test results” has been revised as " Firstly, the results of GSADF test ".

(38) On Page 27, line 535, “Second” has been revised as " Secondly ".

(39) On Page 28, line 540, “Bubbles in 2020 thanks to” has been revised as " Bubbles in 2020 were formed from ".

(40) On Page 28, line 542, “Third” has been revised as " In addition ".

(41) On Page 28, line 545, “Fourth” has been revised as "Finally ".

**Comment 2:** There are several theoretical reasoning which should be more explained:

* Lines 73-74: "If stock prices and dividends [...] in asset price".

**Response:** We have changed “If stock prices and dividends are cointegrated, they share the same stochastic drift, and there is no bubble in asset price.” to “If first differences of stock prices have a stationary mean and/or stock prices are cointegrated with dividends, there would be evidence against the existence of rational bubbles.”

The theoretical reasoning is explained as follow (the details of theoretical model are in section 2.1 of our paper):

Of particular interest to researchers in this area are “rational bubbles”. The concept of rational bubble can be illustrated by financial present value theory, where the real price of an asset is assumed to be equal to the present value of relevant fundamentals and a bubble component that grows in expectation at the real interest rate, and investors are assumed to have rational expectations. Under these assumptions investing in the asset can be a rational choice for investors even though its current observed price is higher than the price level that is justified by relevant fundamentals.

Diba and Grossman (1988) implement stationarity tests for explosive rational bubbles in stock prices using a model that assumes a constant discount rate, but that allows unobservable variables to affect market fundamentals and also allows different valuations of expected capital gains and expected dividends. If the first differences of the unobservable variables and the first differences of dividends are stationary (in the mean) and first differences of stock prices are stationary, then the model implies that rational bubbles do not exist. If the levels of the unobservable variables and the first differences of dividends are stationary, and stock prices and dividends are cointegrated of order (1,1), then rational bubbles do not exist.

Diba and Grossman point out evidence that first differences of stock prices have a stationary mean and/or evidence that stock prices are cointegrated with dividends would be evidence against the existence of rational bubbles. Please refer to article “Explosive Rational Bubbles in Stock Prices?” (Diba and Grossman, 1988).

* Lines 77-78: "Through Dickey-Fuller [...] no bubbles".

**Response:** In page 4, line 78, We have changed “Through Dickey-Fuller test, they found that both dividends and stock prices were stationary in differences, indicating no bubbles.” to “They applied the Dickey-Fuller (DF) test to the price and dividend series in levels and first differences, then found that both stock prices and dividends were stationary in first differences. Since differencing an explosive autoregressive process does not lead to a stationary process, a rejection from the DF test for the first difference of the price and dividend series, suggests that no rational bubble exists.”

The detail theoretical reasoning of this point is the same as the last point, so we won't repeat it here.

* Lines 87-89: This sentence does not make sense.

**Response:** According to the reviewer’s comment, we have deleted the sentence“The initial popular method is the two-regime Markov-switching unit root test [16], which considers the expanding and collapsing periods of bubbles as two different regimes and allows the bubble to switch between two regimes.”. With this operation, we delete the corresponding reference [16]: Van Norden S. Regime switching as a test for exchange rate bubbles. J. Appl. Econom. 1996; 11(3): 219-251. doi: 10.1002/(SICI)1099-1255(199605)11:3<219::AID-JAE394>3.0.CO;2-S.

* Lines 102-103: This sentence does not make sense.

**Response:** Considering the Reviewer’s suggestion, we have changed the sentence “on the basis of PWY, they used right recursive unit root test with flexible window width, namely Generalized Sup ADF, to test the existence of bubbles” to “namely Generalized Sup ADF with flexible window width”.

**Comment 3:** The statements included in the Subsection "Model specification" should be more supported by theoretical literature because this paper introduces the novelty of the ratio ln(p)/ln(d) and this advices to relate this indicator to other parameters involved in the analysis of the necessary and sufficient conditions for the existence of bubbles. To do this, the authors may consult the following references:

**Response:** Thank you very much for your advice on Model specification. The references that you recommend discuss an explicit necessary and sufficient condition on the dividend stream of a publicly traded company, under which the price of the company's share is equal to the present value of the future dividends that will accrue to it. These researches are very valuable. So, we have cited these references in our paper. The details are as below:

* Sethi, S.P.; Derzko, N.A.; Lehoczky, J.P. Mathematical analysis of the Miller-Modigliani theory. Operations Research Lett. 1982, 1 (4), 148–152.

**Response:** We have cited this reference in page 7 line 143 in our paper, and this reference is listed in the reference list as number [35].

* Sethi, S.P.; Derzko, N.A.; Lehoczky, J.P. A stochastic extension of the Miller-Modigliani framework. Mathematical Finance 1991, 1 (4), 57–76.

**Response:** We have cited this reference in page 7 line 143 in our paper, and this reference is listed in the reference list as number [34].

*Sethi, S.P.; Derzko, N.A.; Lehoczky, J.P. When does the share price equal the present value of future dividends? Economic Theory 1996, 8, 307–319.

**Response:** We have cited this reference in page 7 line 143 in our paper, and this reference is listed in the reference list as number [33].

* Cruz Rambaud, S. (2013). Arbitrage Theory with State-Price Deflators, Stochastic Models, 29:3, 306-327.

**Response:** This reference developed some mathematical results for the existence of asset price bubbles, which is consistent with the martingale analysis of financial markets. More specifically, this article studies the relation between the divergence of the sum of dividend-price ratios and the absence of bubbles. So, we have cited this reference in page 9 line 175 in our paper, and this reference is listed in the reference list as number [37].

Reviewer #4:

**Comment 1:** May the authors should take a look at the following reference:

\bibitem{}M.~Fern{\'{a}}ndez-Mart{\'{i}}nez, M.A. S{\'{a}}nchez-Granero, M.J.{Mu{\~{n}}oz Torrecillas}, and B.~McKelvey, \emph{{A COMPARISON OF THREE HURST EXPONENT APPROACHES TO PREDICT NASCENT BUBBLES IN S{\&}P500 STOCKS}},Fractals \textbf{25} (2017), no.~1,

where the transition from efficient market behavior (EMB, hereafter) to the beginning of a bubble in the S&P500 index was explored. With this aim, another technique, based on the self-similarity exponent, was applied. In fact, they found that the higher (resp., the lower) the self-similarity index of the series, the higher (resp., the lower) the mean of the price changes, and hence, the better (resp., the worse) the performance of that stock. As such, the beginning of the transition from EMB to herding behavior could be identified.

**Response:** The reference you recommend explored whether there are some connections between the self-similarity exponent of a stock (as a Herding Behavior indicator) and the stock’s future performance under the assumption that the HB will last for some time. The findings of this reference are significant. We have cited this reference in page 25 line 497 in our paper, and this reference is listed in the reference list as number [41].

We tried our best to improve the manuscript according to the reviewer’s suggestion. Again, we appreciate for the Editors and Reviewer’s time and help for improving the quality of our manuscript. We hope that the correction will meet with approval. If you have any question about this paper, please don’t hesitate to let me know.

Looking forward to hearing from you.

Kind regards,

Ge Li

Corresponding author: Ming Xiao

E-mail address: blue_ridge111@yahoo.com
